# Homeostatic Regulation of Marginal Zone B Cells by Invariant Natural Killer T Cells

**DOI:** 10.1371/journal.pone.0026536

**Published:** 2011-10-26

**Authors:** Xiangshu Wen, Jun-Qi Yang, Peter J. Kim, Ram Raj Singh

**Affiliations:** 1 Autoimmunity and Tolerance Laboratory, Division of Rheumatology, Department of Medicine, David Geffen School of Medicine at University of California Los Angeles, Los Angeles, California, United States of America; 2 University of Cincinnati College of Medicine, Cincinnati, Ohio, United States of America; 3 Jiangsu Institute of Parasitic Diseases, Wuxi, Jiangsu, China; 4 Department of Pathology and Laboratory Medicine, University of California Los Angeles, Los Angeles, California, United States of America; 5 Jonsson Comprehensive Cancer Center, University of California Los Angeles, Los Angeles, California, United States of America; Karolinska Institutet, Sweden

## Abstract

Marginal zone B cells (MZB) mount a rapid antibody response, potently activate naïve T cells, and are enriched in autoreactive B cells. MZBs express high levels of CD1d, the restriction element for invariant natural killer T cells (iNKT). Here, we examined the effect of iNKT cells on MZB cell activation and numbers *in vitro* and *in vivo* in normal and autoimmune mice. Results show that iNKT cells activate MZBs, but restrict their numbers *in vitro* and *in vivo* in normal BALB/c and C57/BL6 mice. iNKT cells do so by increasing the activation-induced cell death and curtailing proliferation of MZB cells, whereas they promote the proliferation of follicular B cells. Sorted iNKT cells can directly execute this function, without help from other immune cells. Such MZB regulation by iNKTs is mediated, at least in part, via CD1d on B cells in a contact-dependent manner, whereas iNKT-induced proliferation of follicular B cells occurs in a contact- and CD1d-independent manner. Finally, we show that iNKT cells reduce ‘autoreactive’ MZB cells in an anti-DNA transgenic model, and limit MZB cell numbers in autoimmune-prone (NZB×NZW)F1 and non-obese diabetic mice, suggesting a potentially new mechanism whereby iNKT cells might regulate pathologic autoimmunity. Differential regulation of follicular B cells versus potentially autoreactive MZBs by iNKT cells has important implications for autoimmune diseases as well as for conditions that require a rapid innate B cell response.

## Introduction

B cells in the spleen anatomically localize in the follicles and marginal zone and are called follicular B cell (FoB) and marginal zone B cell (MZB), respectively [1], [2]. The MZBs exhibit unique characteristics not shared by FoBs. Their location in the spleen helps MZBs mount a rapid antibody response to blood-borne antigens independently of T cell help [3]. MZBs continuously shuttle between marginal zone and lymphoid follicles to transport antigens to follicular dendritic cells [4] and potently activate naïve CD4^+^ T cells and quickly differentiate into plasma cells [5]. Rapid and efficient regulatory mechanism(s) must exist to curb such prompt and vigorous responsiveness of MZBs to avoid unnecessary immune activation.

MZBs are phenotypically distinct from other B cells and are identified as CD21^hi^ CD35^hi^ CD23^low^ CD9^+^ IgD^low/−^ IgM^hi^ cells [1], [2]. MZBs are also distinct from other B cell subsets through their high expression of CD1d [6]. Since CD1d presents lipid antigens to invariant natural killer T (iNKT) cells [7], [8], [9], MZBs have been proposed to be important activators of iNKT cells. However, it is unclear whether iNKT cells, in turn, activate or regulate MZBs in a way that is different from their interactions with other B cell populations.

iNKT cells rapidly respond to glycolipid antigens, such as α-galactosylceramide (αGalCer) [10]. These cells trans-activate a variety of other cells, including NK cells, conventional T cells, and B cells [11], [12], [13], [14]. For example, iNKT cells enhance peripheral blood B cell proliferation [15] and enhance immunoglobulin production against T-dependent and T-independent antigens and pathogens [15], [16], [17], [18]. It is unclear whether iNKT cells interact differently with MZBs that express particularly high levels of CD1d compared to FoBs.

MZB cells have several features required to break T cell tolerance. For example, MZB cells can act as robust APCs [5] and can be easily activated by dendritic cells [19]. In fact, several studies have linked MZB cell abnormalities to the development of autoimmune diseases [20], [21], [22]. MZB cells expand in the non-obese diabetic (NOD) mouse model of type 1 diabetes (T1D) prior to the onset of disease at an early age when autoreactive T cells begin to appear [23], [24]. MZB cells are also increased in lupus mice [25], [26], and B cells bearing anti-self B cell receptors are enriched in the marginal zone of spleens [20], [27]. Thus, understanding mechanisms of MZB cell homeostasis will have important implications for understanding autoimmune diseases.

In this article, we examined the effects of iNKT cells on the activation, proliferation and frequency of major B cell subsets in the spleen, MZB and FoB, *in vitro* and *in vivo* in normal and autoimmune-prone mice. Our results show that while iNKT cells activate both MZB and FoB, they selectively curtail the proliferation of MZBs and promote their activation-induced cell death (AICD). These results indicate a role of iNKT cells in regulating the homeostasis of MZBs. Such regulation of MZBs might be an important mechanism of controlling autoimmune diseases, since iNKT cells reduce ‘autoreactive’ anti-DNA MZBs and limit MZBs in lupus-prone NZB/NZW F1 and autoimmune diabetes-prone NOD mice. These data, along with our recent report describing the role of iNKT cells in inhibiting autoantibody production [28], have important implications for the development of iNKT cell-based therapy in autoimmune diseases.

## Materials and Methods

### Ethics Statement

All of the scientific data were obtained using high scientific, technical, and ethical standards. All animal experiments were approved by UCLA Office of Research Oversight Chancellor's Animal Research Committee under protocol ARC # 2005-155.

### Mice

BALB/cJ, C57BL/6, NOD/ShiLtJ, NZB and NZW mice were obtained from the Jackson Laboratory (Bar Harbor, ME) and bred locally. R4A-γ2b^Tg^ mice that have increased numbers of IgG2b anti-dsDNA Ab B cells [20] were provided by Dr. B. Diamond. Vα14^Tg^ [29] and CD1d^−/−^mice [26] were provided by Dr. A. Bendelac and Dr. L. Van Kaer, respectively. CD1d^−/−^ BWF1 mice were generated by introgressing CD1d-null allele onto NZB and NZW backgrounds for 10 and 12 generations, respectively, and then intercrossing them [30]. The mouse genotype was confirmed by PCR and their phenotype was confirmed by flow cytometry using anti-CD1d antibody (1B1) for CD1d^−/−^ mice and mCD1d/PBS-57 tetramer for Vα14^Tg^ and CD1d^−/−^ mice [30].

### Reagents

The mCD1d/PBS-57 tetramer was obtained from the NIH Tetramer Core Facility (Emory, GA). Anti-CD93 antibody (AA4.1) was from eBiosciences; anti-IgM antibody was from Jackson ImmunoResearch; all other antibodies were from BD Biosciences (San Diego, CA).

### Flow cytometry

Spleen cell suspensions were prepared in staining buffer (0.5% BSA, 0.09% sodium azide) after red blood cell lysis by Tris-NH_4_Cl (pH 7.2). Cells were incubated with anti-CD16/32 (2.4G2) to block FcγRII/III, followed by staining with conjugated mAbs for mouse antigens, including CD1d (1B1), TCRβ (H57-597), CD86 (GL1), CD69 (H1.2F3), CD95 (Jo2), IgM (μchain specific), CD19 (1D3), B220 (RA3/6B2), CD21 (7G6), CD23 (B3B4), CD9 (KMC8), active form of caspase3 (C92-605), 7AAD, and mCD1d/PBS-57 tetramer. Flow cytometry analysis was performed using FACSCalibur or FACScan (Becton Dickinson). Data were analyzed using FlowJo software (Ashland, OR) with lymphocyte gate, based on forward and side scatter.

### Immunohistochemistry

Spleen was embedded in tissue freezing medium (Fisher Scientific, Pittsburgh, PA) and frozen in liquid nitrogen. Frozen tissue blocks were sectioned using HM550 cryostat (Mikron, San Marcos, CA) and 7 µm sections mounted on Superfrost plus slides (Fisher Scientific). Slides were dried at room temperature (RT), fixed with cold acetone for 2 min, dried at RT and then rinsed three times with PBS. Slides were incubated in a humidified chamber with blocking buffer (20 µg/ml CD16/CD32, 10% normal mouse serum, 2.5% BSA and 0.1% tween-20 in PBS) for 30 min at RT. Blocked sections were stained with biotin-IgM (μchain specific, Jackson ImmunoResearch) and FITC-conjugated MOMA1 (CD169) (AbD Serotec, Raleigh, NC) at 10 µg/ml on RT for 2 h and then rinsed 5-times with PBST (0.1% Tween20 in PBS). Slides were further incubated with 2 µg/ml of APC-conjugated streptavidin (BD Biosciences) for 1 h at RT and then rinsed 5-times with PBST. Stained slides were mounted with crystal mount (Biomeda, Foster City, CA) and images captured using LEICA DM IRB (Meyer instruments, Houston, TX). Images were analyzed using Leica and Photoshop software.

### *In vivo* immunization

Mice were immunized i.p. with 4 µg of αGalCer or vehicle. Their spleen was collected at various time points after injection for analyses.

### Purification of T, B and iNKT cells

Spleen cells were incubated with anti-CD90 or anti-CD19 microbeads (Miltenyi Biotec, Auburn, CA) for 20 min at 4°C to purify T cells and B cells, respectively, using AutoMACS (Miltenyi Biotec). The purity of T cells and B cells ranged from 82–86% and 97–98%, respectively. The iNKT cells were sorted as TCRβ^+^mCD1d/PBS-57 tetramer^+^ cells and conventional T cells sorted as TCRβ^+^ mCD1d/PBS-57 tetramer^−^ cells using FACSAria (Becton Dickinson). The sorted cells were collected in complete RPMI-1640 medium and checked for their purity (>98%).

### Cell culture

Spleen cells at 2×10^6^ per ml in complete RPMI-1640 medium were added to 24-well polystyrene, flat-bottom tissue culture plates (Corning, Costar, NY) and cultured at 37°C in a humidified 5% CO_2_ incubator. Purified B cells (1–2×10^6^ per ml) were cultured with or without LPS (10 µg/ml) and αGalCer (50–100 ng/ml). Sorted T cells or iNKT cells were added to these cultures at 0.5–1×10^6^ cells per ml. Cells were collected at the indicated time points, stained with indicated antibodies, and analyzed by flow cytometry.

### B cell proliferation assay

Purified B cells in complete RPMI-1640 medium were labeled with carboxyfluorescein diacetate succinimidyl ester (CFSE) with CellTraceTM CFSE cell proliferation kit (Invitrogen), following the manufacturer's instructions. LPS, αGalCer, and sorted T cells or iNKT cells were added to cultures, as described above. Cells were collected at the indicated time points, stained with anti-CD19 (PerCP-Cy5.5), anti-CD21 (PE), and anti-CD23 (biotin) antibodies, followed by APC-conjugated streptavidin), and analyzed by flow cytometry.

### Statistical analysis

Descriptive statistics are expressed as the mean ± SE values. Comparisons between groups were performed using two-tailed Student *t* test or Mann-Whitney *U* test, and a *p* value of <0.05 was considered significant.

## Results

### iNKT cells activate MZBs, but restrict their numbers *in vivo* and *in vitro*

**Figure 1:**
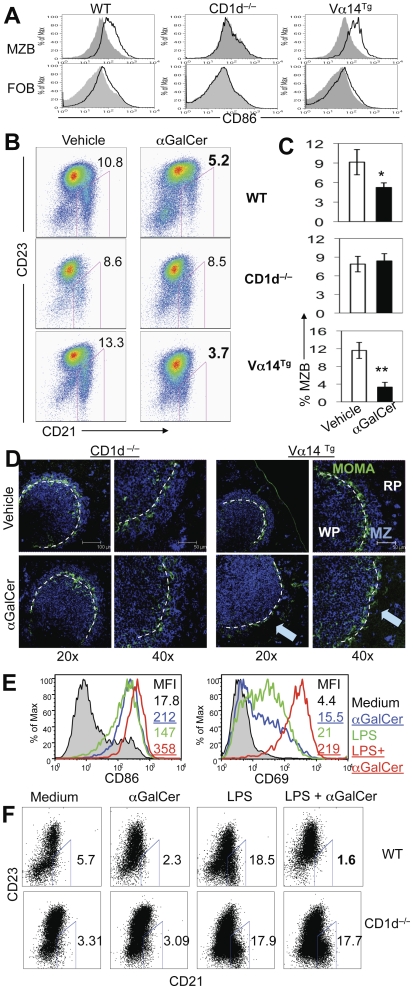
Effect of αGalCer treatment on activation and numbers of MZBs *in vivo* and *in vitro*. **A–D**. Four-mo-old female CD1d^−/−^, WT and Vα14^Tg^ BALB/c mice were injected i.p. with 4 µg of αGalCer or vehicle. Their spleens were harvested 3–4 d later and analyzed by flow cytometry and immunohistochemistry for MZBs. Results using three mice per group from one representative of at least three independent experiments are shown. **A**. CD86 expression on gated CD19^+^CD21^hi^CD23^−/low^ (MZBs) and CD19^+^CD21^+^CD23^+^ (FoBs) in αGalCer (thick line) or vehicle (shaded area) injected mice. **B**. MZBs (CD21^hi^CD23^−/low^) were analyzed on gated CD19^+^ lymphocytes. MZB frequency is expressed as % of CD19^+^ lymphocytes in representative dotplots (**B**) and as the mean±SE from three each of vehicle or αGalCer-injected WT, CD1d^−/−^ and Vα14^Tg^ mice (**C**). A significant reduction of MZBs was found in αGalCer-treated WT (*p<0.05) and Vα14^Tg^ mice (**p<0.01), but not in CD1d^−/−^ mice. **D**. Frozen spleen sections from αGalCer or vehicle treated mice were stained for APC-IgM (blue) and FITC-MOMA1 (green). Confocal images show IgM^+^ cells (blue) in the marginal zone between red pulp and MOMA-1 (green) in CD1d^−/−^ and vehicle-injected Vα14^Tg^ mice. IgM^+^ cells in the marginal zone are reduced in αGalCer-treated Vα14^Tg^ mice (as indicated by a blue arrow). MZ, marginal zone; RP, red pulp; WP, white pulp. 20× and 40× magnification. **E**, **F**, Spleen cells (2×10^6^ cells per ml) from 3-mo-old BALB/c mice were cultured with or without LPS in the absence or presence of αGalCer (100 ng/ml). Results represent five independent experiments, each time using cells from one mouse per group. **E**. Expression of CD69 and CD86 are shown on gated MZBs (CD19^+^CD21^hi^CD23^−/low^) in spleen cells cultured with medium alone (shaded area), αGalCer (blue line), LPS (green line) or LPS+αGalCer (red line) for 24 h. The MFI of CD69 and CD86 are shown. **F**. Spleen cells from WT and CD1d^−/−^ BALB/c mice were cultured without or with LPS and/or αGalCer for 72 h. MZB cells are expressed as % of mature B cells (AA4.1^−^ IgM^+^).

To determine the effect of iNKT cell activation on different B cell subsets, we injected αGalCer i.p. in BALB/c mice, including WT, Vα14^Tg^ mice that express the canonical TCR α chain Vα14-Jα18 of iNKT cells [29], and CD1d^−/−^ mice that have no iNKT cells [26], and analyzed activation markers on freshly isolated spleen cells. αGalCer treatment increased CD86 expression on both MZBs and FoBs from WT or Vα14^Tg^ mice, but not from CD1d^−/−^ mice (**Fig. 1A**). Unexpectedly, however, *in vivo* αGalCer treatment resulted in a marked reduction in MZB frequency and marginal zone IgM^+^ B cells in Vα14^Tg^ and WT mice, but not in CD1d^−/−^ mice (**Fig. 1B–D**, and data not shown).

*In vitro* studies showed that αGalCer was as strong as LPS in activating MZBs, and acted synergistically with LPS (**Fig. 1E**). However, addition of αGalCer to spleen cell cultures reduced the proportions of MZBs (**Fig. 1F**, upper panel). Total MZB numbers were also reduced in the presence of αGalCer (LPS 5.9×10^5^; LPS+αGalCer 0.3×10^5^). Similar results were obtained using spleen cells from Vα14^Tg^ mice (data not shown), whereas no change in MZB cells were seen using spleen cells from CD1d^−/−^ mice (**Fig. 1F**, lower panel).

**Figure 2:**
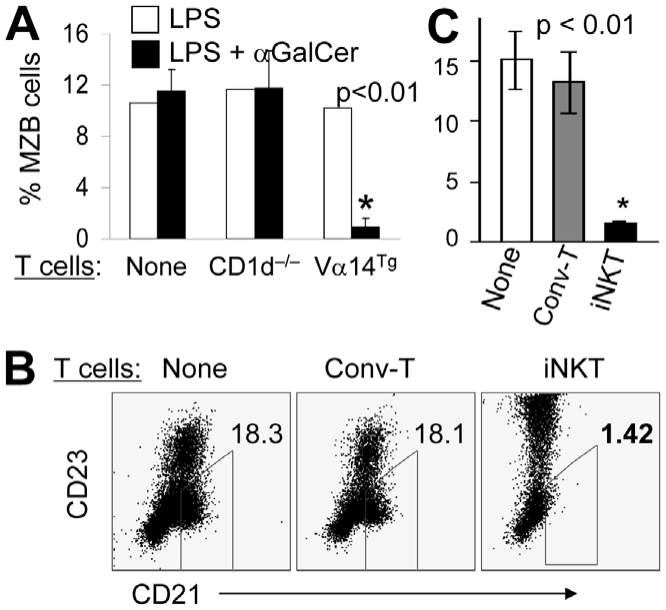
iNKT cells can directly inhibit MZBs. **A**. (left bars) αGalCer does not directly affect B cells. Purified B cells (1.5×10^6^ cells per ml) from WT BALB/c mice were cultured alone and stimulated with LPS in the presence or absence of αGalCer for 68 hours. (Middle and right bars) T cells are sufficient to execute αGalCer-induced regulation of MZB numbers. Purified B cells from WT BALB/c mice were co-cultured with purified CD90^+^ T cells (7.5×10^5^ cells per ml) from CD1d^−/−^ or Vα14^Tg^ mice, with LPS and/or αGalCer. MZBs were analyzed as IgM^+^CD21^hi^CD23^−/low^ by flow cytometry and expressed as the mean ± SE (*p<0.01; n = 3 mice per group) from three independent experiments. **B**, **C**. Sorted iNKT cells reduce MZB cell numbers. Spleen cells from Vα14^Tg^ mice were sorted as iNKT cells (TCRβ^int^ mCD1d-PBS57 tetramer^+^) or conventional T cells (TCRβ^+^ mCD1d-PBS57 tetramer^−^; Conv-T). The sorted T cells (5×10^5^) were co-cultured with purified B cells (1×10^6^) from WT mice in presence of LPS and αGalCer for 2–3 d. MZBs were analyzed as CD19^+^CD21^hi^CD23^low^ cells, and summarized in the bar diagram (**C**) as the mean ± SE percentage of CD19^+^ MZBs (*p<0.01; n = 3 mice per group). Data shown represent three independent experiments.

Next, we observed that the reduction of MZB cells in the presence of αGalCer was not due to its direct binding/effect on MZB cells (**Fig. 2A**, left bars), but was mediated by T cells as the co-culture of B cells with purified T cells from WT mice or Vα14^Tg^ mice [that have ∼50% T cells expressing iNKT cell TCR], but not from CD1d^−/−^ mice, reduced MZBs (**Fig. 2A**, middle and right bars). Strikingly, the presence of sorted iNKT cells alone was sufficient to reduce MZBs, whereas purified conventional T cells had no effect on MZB frequency (**Fig. 2B,C**). The sorted iNKT cells, in the absence of other T cell subsets, also markedly enhanced CD23 expression. These data clearly show that while iNKT cells can activate MZBs and FoBs, they can directly and selectively restrict the MZB population.

### iNKT cells promote AICD of MZBs *in vitro* and *in vivo*

**Figure 3:**
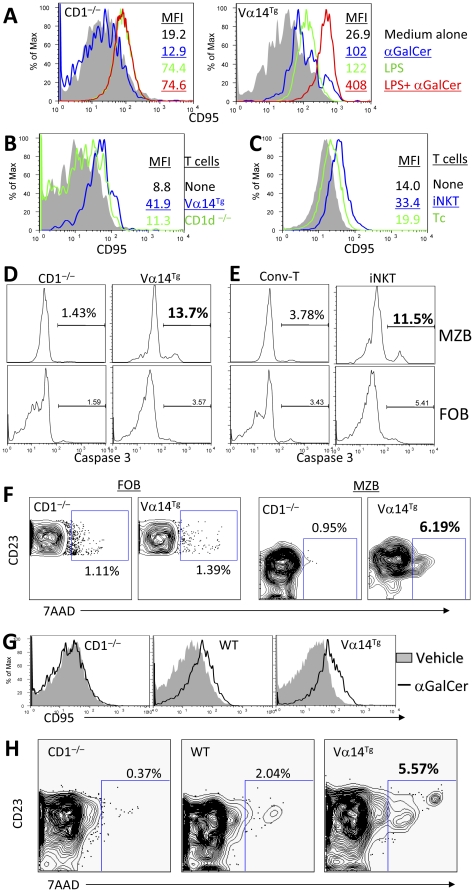
Effect of iNKT cells on apoptosis markers and AICD of MZBs *in vitro* and *in vivo*. **A–C**. iNKT cells increase CD95 expression on MZBs *in vitro*. (**A**) Spleen cells (2×10^6^ cells per ml) from CD1d^−/−^ or Vα14^Tg^ BALB/c mice were cultured with medium alone (shaded area), αGalCer (blue line), LPS (green line) or LPS+αGalCer (red line) for 3 d. Representative histograms show the expression of CD95 on MZBs (CD19^+^CD21^hi^CD23^−/low^). Similar results were obtained when spleen cells from WT mice were used in place of Vα14^Tg^ mice. Results are representative of more than five independent experiments, each time using one mouse per group. (**B**) B cells purified from WT BALB/c spleen were cultured alone (shaded area) or with CD90^+^ T cells from CD1d^−/−^ mice (green line) or Vα14^Tg^ mice (blue line) in the presence of LPS and αGalCer for 2 d. CD95 expression is shown on gated MZBs from one representative of two experiments, each time using B cells pooled from two mice and T cells pooled from three mice. (**C**) Purified CD19^+^ B cells from WT BALB/c spleen were cultured alone (shaded area) or with sorted iNKT cells (TCRβ^+^tetramer^+^, blue line) or conventional T cells (TCRβ^+^tetramer^−^, green line; Conv-T) from Vα14^Tg^ mice for 3 d in the presence of LPS and αGalCer. CD95 expression is shown on gated MZBs from an experiment using pooled cells from 3–4 mice per group. **D**, **E**. iNKT cells increase active caspase3 expression in MZBs. CD19^+^ B cells (2×10^6^ cells per ml) from WT BALB/c mice were cultured with LPS and αGalCer. To these wells, we added CD90^+^ T cells (1×10^6^ cells per ml) from Vα14^Tg^ or CD1d^−/−^ BALB/c mice (**D**) or sorted iNKT cells (TCRβ^+^tetramer^+^) or conventional T cells (Conv-T; TCRβ^+^tetramer^−^) at 1×10^6^ cells per ml (**E**). After 2–3 d of cultures, cells were analyzed for the expression of active caspase3 on MZBs and FoBs. Compared to cultures containing CD1d^−/−^ or tetramer^−^ T cells, cultures containing Vα14^Tg^ T cells or sorted iNKT cells had 3–9-fold higher expression of active caspase3 on MZBs but not on FoBs. Results represent two independent experiments, each using cells pooled from 2–3 mice per group. **F**. iNKT cells induce apoptosis of MZBs *in vitro*. CD19^+^ B cells from WT BALB/c mice were cultured with CD90^+^ T cells from Vα14^Tg^ or CD1d^−/−^ BALB/c mice for 48 h, as in panel **D**, and expression of 7AAD was analyzed on FoBs or MZBs. Whereas iNKT cells (T cells from Vα14^Tg^ mice) did not increase the proportion of 7AAD^+^ FoBs, they increased the proportion of 7AAD^+^ apoptotic MZBs by 6–7-fold compared to cultures that had no iNKT cells. Results represent two independent experiments, each using pooled cells from 2–5 mice per group. **G**. Enhanced CD95 expression on MZBs upon activation of iNKT cells *in vivo*. 4-mo-old animals were injected i.p. with 4 µg of αGalCer (thick line) or vehicle (shaded area). Freshly isolated spleen cells were analyzed for CD95 expression on MZBs at 3 d post-injection. Data represent three independent experiments, each time using one mouse per group. **H**. Increased apoptosis of MZBs upon activation of iNKT cells *in vivo*. Freshly isolated spleen cells from 4–7-mo-old CD1d^−/−^, WT and Vα14^Tg^ mice were analyzed for 7AAD^+^ apoptotic MZBs. Data represent three independent experiments, each time using one mouse per group.

Since iNKT cells markedly activate MZBs yet reduce their numbers, we surmised whether iNKT cells induce AICD of MZBs. Consistent with this hypothesis, addition of αGalCer to spleen cells upregulated the expression of apoptotic marker CD95 (Fas) on MZBs in WT or Vα14^Tg^ mice, but not in CD1d^−/−^ mice (**Fig. 3A**, and data not shown), while LPS upregulated CD95 expression both in WT as well as in CD1d^−/−^ mice. CD95 expression on MZBs increased in the presence of purified CD90^+^ T cells from Vα14^Tg^ mice, but not from CD1d^−/−^ mice in presence of both LPS and αGalCer (**Fig. 3B**). Furthermore, sorted iNKT cells alone could directly increase CD95 expression on MZBs, without any help from other immune cells (**Fig. 3C**). Similar data were obtained, when we examined the effect of activated iNKT cells on active caspase3, an important mediator of apoptosis pathway downstream of Fas, by intracellular staining. Co-culture of B cells in the presence of T cells from Vα14^Tg^ mice or sorted iNKT cells increased caspase3 positive cells among MZBs, but not among FoBs (**Fig. 3D,E**). Finally, whereas activated iNKT cells did not increase apoptotic FoBs, they increased the proportion of apoptotic MZBs by 6-fold (**Fig. 3F**). Similar data were obtained *in vivo*. After a single αGalCer injection, CD95 expression on MZBs as well as the frequency of apoptotic MZBs increased among freshly isolated spleen cells in WT and Vα14^Tg^ mice, but not in CD1d^−/−^ mice (**Fig. 3G,H**). CD95 expression also increased on FoBs in αGalCer-injected WT and Vα14^Tg^ mice, but there was no increase in apoptotic FoBs in these mice (data not shown). Taken together, these data suggest that iNKT cells selectively trigger the AICD of MZBs by enhancing the expression of molecules, CD95 and active caspase3, in the apoptosis pathway. However, iNKT cells also reduced MZBs in *Fas*-mutant MRL-*lpr* mice (not shown in figure), suggesting existence of additional mechanism(s) whereby iNKT cells might regulate MZB cells.

### iNKT cells selectively inhibit the proliferation of MZBs, but not of FoBs

**Figure 4:**
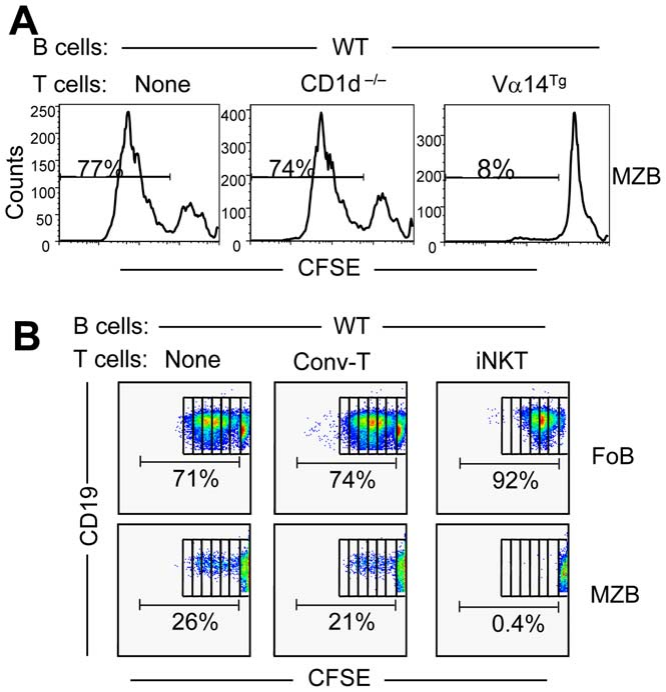
Effect of iNKT cells on the proliferation of FoB versus MZBs. Purified B cells (2×10^6^) from WT BALB/c mice were labeled with CFSE, and cultured alone or co-cultured with CD90^+^ T cells (1×10^6^) from CD1d^−/−^ or Vα14^Tg^ mice for 5 d (**A**) or with sorted conventional T cells (tetramer^−^; Conv-T) or iNKT (tetramer^+^) cells (**B**) for 62–68 h in the presence of LPS and αGalCer. CFSE level was analyzed on MZBs or FoBs on gated CD19^+^ cells. Numbers on the plots represent the % of proliferating cells. Results are representative of three independent experiments, each time using spleen cells pooled from 2–3 mice per group.

In search for additional mechanisms of iNKT cell-mediated inhibition of MZBs, we asked if iNKT cells affect the proliferation of MZBs. CFSE-labeled B cells from WT mice were co-cultured with T cells from CD1d^−/−^ or Vα14^Tg^ mice. Surprisingly, the LPS-induced proliferation of MZBs was profoundly inhibited in cultures containing iNKT cells (**Fig. 4A**). This inhibitory effect was directly mediated by iNKT cells, as sorted tetramer^+^ iNKT cells, but not tetramer^−^ conventional T cells, completely suppressed the proliferation of MZBs (**Fig. 4B**). In contrast, FoBs robustly proliferated in the presence of iNKT or conventional T cells. Thus, iNKT cells have disparate effects on the proliferation of FoBs versus MZBs.

### iNKT cell-mediated inhibition of MZB proliferation is partly contact- and CD1d-dependent

**Figure 5:**
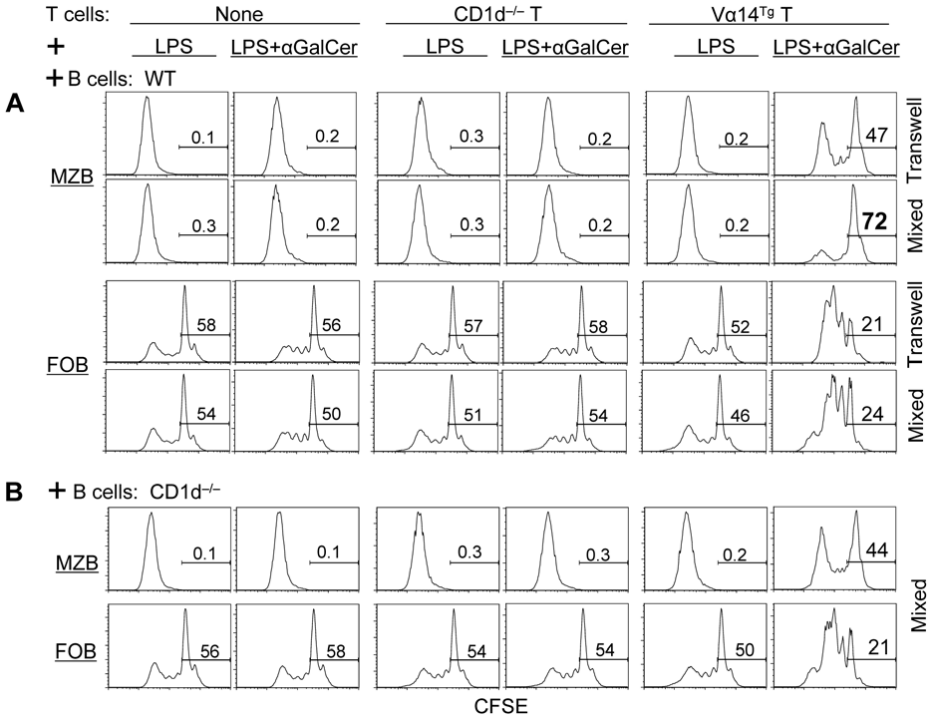
Role of cell–cell contact and CD1d in iNKT cell-mediated inhibition of MZB cells. Purified T cells from CD1d^−/−^ or Vα14^Tg^ BALB/c mice were co-cultured with CFSE-labeled CD19^+^ B cells from WT or CD1d^−/−^ mice in the presence of LPS with or without αGalCer for 4 days. Cells were then analyzed for CFSE dilution on gated MZBs (TCRβ^−^CD21^hi^CD23^−/low^) or FoBs (TCRβ^−^CD21^+^CD23^+^). Results are representative of two independent experiments, each time using spleen cells pooled from 2–3 mice per group.

To investigate mechanisms whereby iNKT cells inhibit MZB proliferation, we first conducted trans-well culture experiments, where CFSE-labeled B cells from WT mice were cultured together or separated in transwells with T cells (**Fig. 5A**). Results show that LPS-induced proliferation of MZBs was inhibited by iNKT cells in both transwells and mixed cultures, suggesting a role of both cell-cell contact and cytokine-dependent mechanisms in iNKT cell-mediated inhibition of MZB cells. However, MZB cell proliferation was more profoundly inhibited in mixed cultures than in transwells, whereas iNKT cells increased FoB proliferation equally in transwells and mixed cultures. Thus, the efficient inhibition of MZB cells by iNKT cells requires contact between these two cell types.

To investigate mechanisms underlying the contact-dependent inhibition of MZBs by iNKT cells, we determined the role of CD1d that is highly expressed on MZB cells. In co-cultures of Vα14^Tg^ T cells with B cells from WT or CD1d^−/−^ mice, iNKT cells more profoundly inhibited WT MZBs (**Fig. 5A**–MZB, mixed cultures) than CD1d^−/−^ MZBs (**Fig. 5B**), whereas iNKT cell-mediated increase in FoB proliferation was similar in cultures containing WT B cells (**Fig. 5A**–FoB) or CD1d^−/−^ B cells (**Fig. 5B**–FoB). Thus, while iNKT cell-induced FoB proliferation is mediated via soluble factors, iNKT cells inhibited MZBs in a partly CD1d-restricted, contact-dependent manner.

### iNKT cells reduce ‘autoreactive’ MZB cells

**Figure 6:**
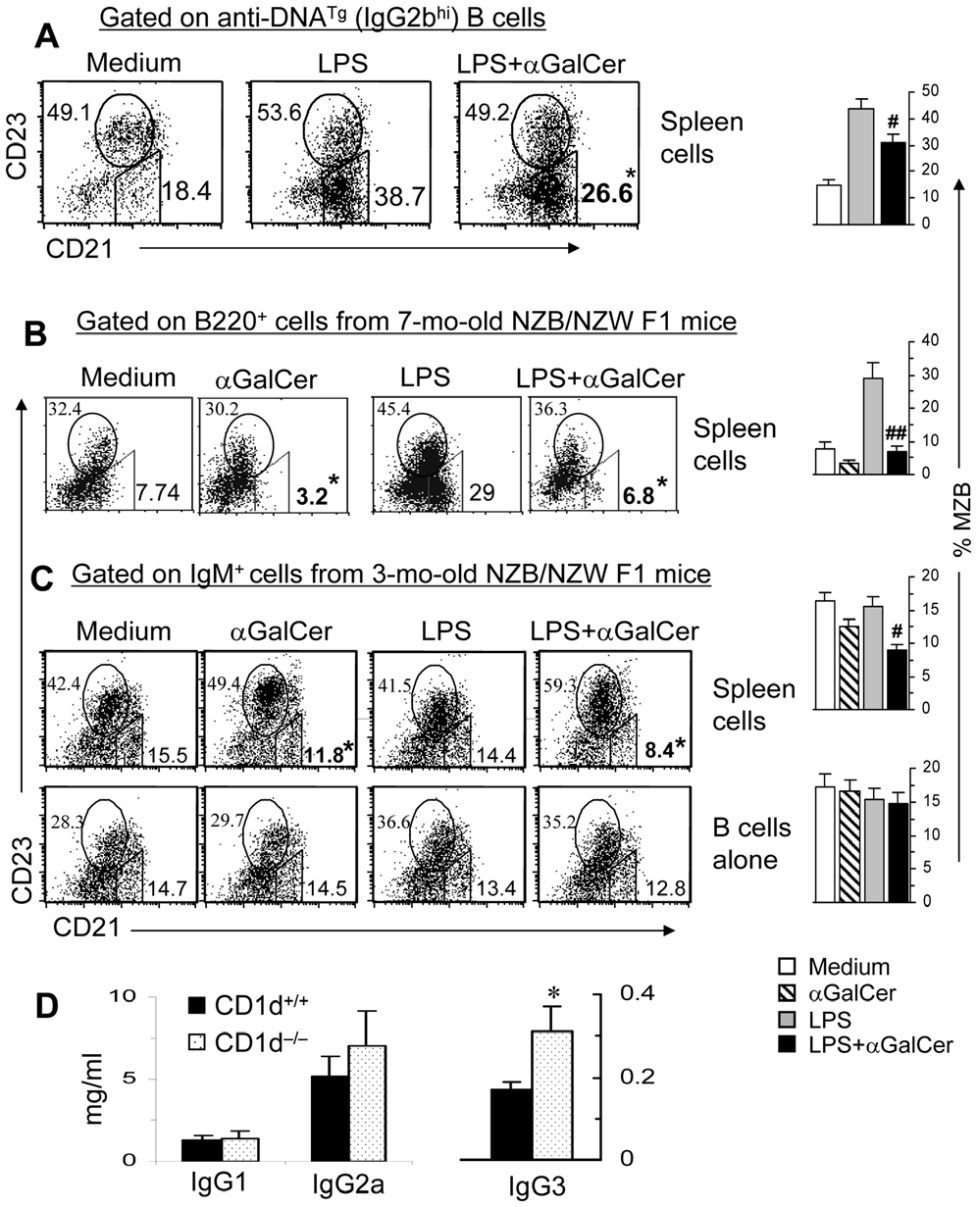
iNKT cells reduce MZB cells in anti-DNA transgenic and lupus-prone mice. (**A**). Spleen cells from 4-mo-old R4A anti-DNA^Tg^ NZW mice were cultured with LPS without or with αGalCer for 48 h, and analyzed for MZBs as the % of anti-DNA (IgG2b^hi^) B cells. (**B**) Spleen cells from 7-mo-old nephritic BWF1 mice were cultured with αGalCer and/or LPS for 65 h, and analyzed for MZBs as the % of B220^+^ lymphocytes. (**C**) Whole spleen cells or purified splenic B cells from 3-mo-old pre-clinical BWF1 mice were cultured with αGalCer and/or LPS for 18 h, and analyzed for MZBs as the % of IgM^+^ lymphocytes. *Note that the addition of αGalCer to spleen cell cultures reduced the proportion of MZB cells. Bar graphs on the right show results expressed as the mean±SE of three independent experiments performed using pooled cells from 3–4 mice per experiment. ^#^P<0.05 ^##^P<0.01, compared to LPS alone cultures. Results represent three independent experiments. (**D**). Serum levels of IgG1, IgG2a and IgG3 isotypes are shown in 8-mo-old CD1d^−/−^ and CD1d^+/+^ BWF1 littermates. *p = 0.04, n = 13 CD1d^−/−^ and 7 CD1d^+/+^ mice, mean ± SE.

The above studies clearly demonstrate the ability of iNKT cells to restrict MZB population in normal BALB/c mice. Since lupus-like autoreactive B cells are enriched in MZB population [20], [27], we asked if iNKT cells can inhibit ‘autoreactive’ MZBs. Whole spleen cells from R4A-γ2b^Tg^ mice that have increased numbers of anti-DNA B cells [20] were cultured with LPS in the presence or absence of αGalCer and MZB cells were enumerated among anti-DNA B cells (IgG2b^hi^-gated cells). As shown in **Fig. 6A**, LPS markedly expanded the anti-DNA MZB cells, which were reduced upon addition of αGalCer. Thus, iNKT cells can specifically regulate autoreactive MZB population.

### iNKT cells reduce MZBs *in vivo* in genetically autoimmune-prone strains of mice

The above studies showing reduction of anti-DNA MZBs in presence of iNKT cells prompted us to ask if iNKT cells will inhibit MZB cells in genetically lupus-prone mice. Results show that addition of αGalCer to spleen cell cultures from diseased 7-mo-old (**Fig. 6B**) or preclinical 3-mo-old NZB/NZW F1 mice (**Fig. 6C**) selectively reduced the frequency of MZB cells. The MZB cell changes were not due to direct binding of αGalCer to CD1d on MZB cells, since adding αGalCer to pure B cells alone had no effect on MZB cell frequency in lupus mice (**Fig. 6C**, lower row). Thus, iNKT cells can regulate MZBs in mice that spontaneously develop autoimmune disease.

Since MZB cells preferentially secrete IgG3 isotype, we asked if the presence of iNKT cells would affect the levels of IgG3 isotype. As shown in **Fig. 6D**, while serum IgG1 and IgG2a isotype levels were similar between the two groups, serum IgG3 levels were higher in CD1d^−/−^ NZB/NZW F1 mice than in CD1d^+/+^ littermates. Taken together, these results as well as data in Fig. 1, 4 and 5 provide evidence that iNKT cells can influence various functions of MZBs including Ig production, activation and proliferation.

**Figure 7:**
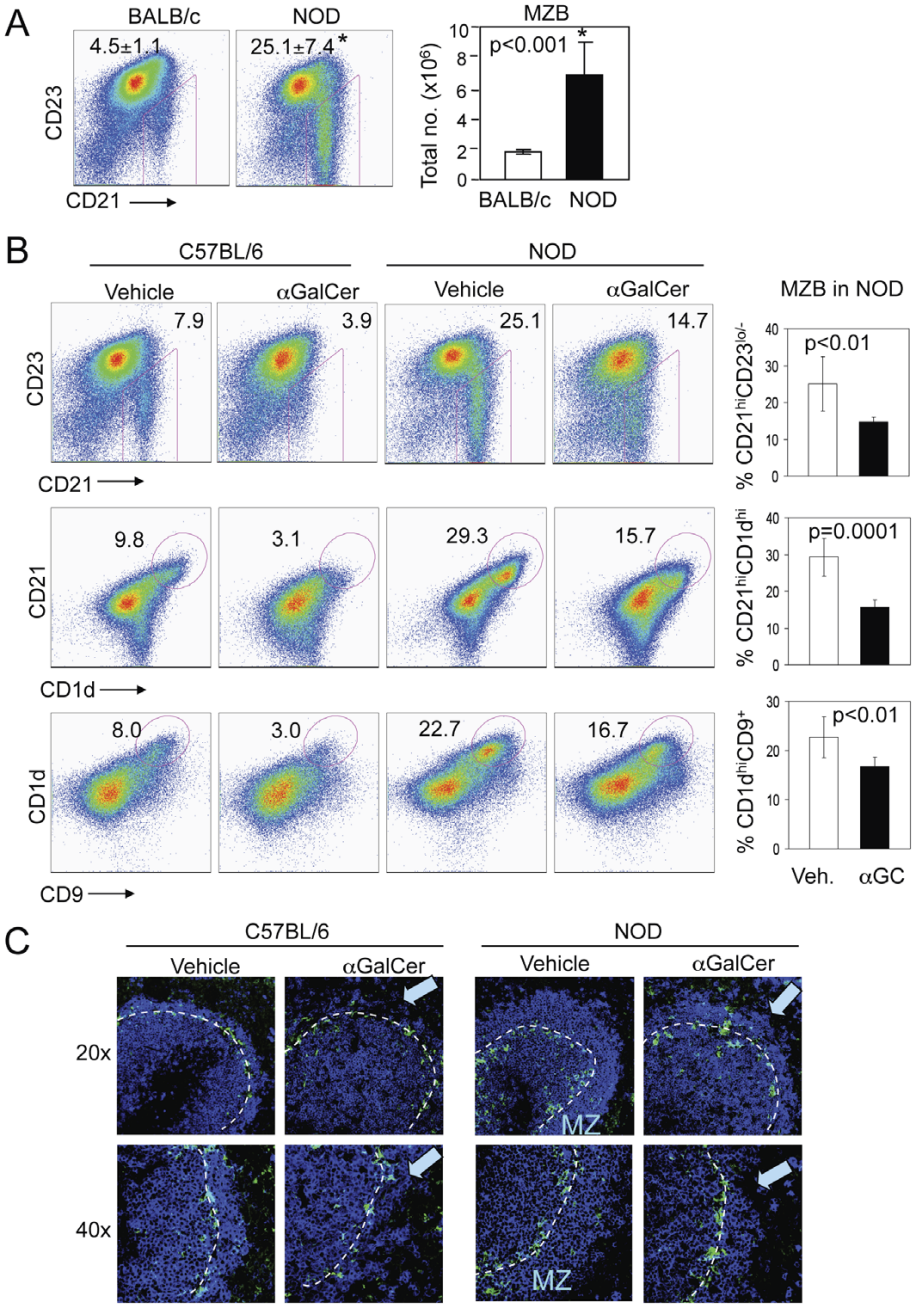
αGalCer treatment limits MZB expansion in NOD mice. **A**. Spleen cells from NOD or normal BALB/c mice were analyzed for MZBs. Comparison of MZB frequency between NOD and BALB/c mice (n = 6 NOD and 3 BALB/c mice, 8–10-week-old females). Numbers on dot plots indicate MZBs (CD21^hi^CD23^−/low^) as the mean ± S.E. % of CD19^+^ cells (*p<0.001). Bar diagram shows the mean ± S.E. absolute numbers of MZBs per spleen (*p<0.001). (**B, C**) Spleens were harvested from 2-mo-old NOD or C57BL/6 mice at 3 d after a single i.p. injection with αGalCer or vehicle (n = 6 mice per group). **B**, Cells were analyzed for MZBs as CD21^hi^ CD23^−/low^ CD1d^hi^ CD9^+^ cells on gated CD19^+^ B cells. Numbers on dotplots indicate MZBs as % of CD19^+^ B cells. Bar diagrams show the mean ± S.E. % MZBs in NOD mice. Compared to vehicle-injected mice, αGalCer-treated NOD mice had a significant reduction in MZBs, defined as CD19^+^CD21^hi^CD23^−/low^ (p = 0.007), CD19^+^CD21^hi^CD1d^hi^ (p = 0.0001), CD19^+^CD1d^hi^CD9^+^ (p = 0.009). **C**, Frozen spleen sections were stained for APC-IgM (blue) and FITC-MOMA1 (green). Confocal images at 20× and 40× magnification show the reduced thickness of marginal zone (MZ) IgM^+^ cells (blue) in αGalCer-treated mice (as indicated by blue arrows). Data represent three independent experiments.

Our results thus far show that iNKT cell effects on MZBs were particularly pronounced under condition, such as *in vitro* LPS exposure, that is known to cause MZB expansion [1]. Consequently, we asked if iNKT cell activation will restrict MZBs *in vivo* in animals that spontaneously develop marked MZB expansion. Results show that NOD mice that have reduced numbers of iNKT cells [31] exhibit increased MZB cell proportions and numbers and increased marginal zone B cell area as compared to normal BALB/c (**Fig. 7A**) and B6 mice (**Fig. 7B**), as also reported previously [23], [24]. To further test the role of this association between reduced iNKT cells and increased MZBs, we treated NOD mice with αGalCer that expands iNKT cells and prevents the development of diabetes [32], [33]. As shown in **Fig. 7B**, αGalCer treatment, as compared to vehicle injection, resulted in a significant reduction in the frequency of MZBs in NOD mice. B cells were also reduced in the marginal zones of spleens in αGalCer-treated NOD mice (**Fig. 7C**). Thus, iNKT cell activation can reduce MZB cells *in vivo* in autoimmune mice that have expanded MZB population.

## Discussion

We report that while iNKT cells activate the two major subsets of B cells in the spleen, they selectively reduce MZBs *in vitro* and *in vivo*. This reduction in MZBs is at least in part due to their increased AICD as well as due to reduced proliferation in a partly CD1d-restricted, contact-dependent manner. In contrast, iNKT cells promote the proliferation and expansion of FoB cells via soluble factors. Thus, iNKT cells interact differently with two major B cell subsets in the spleen, whereby iNKT cells selectively regulate MZB homeostasis.

We demonstrate the iNKT cell-mediated inhibition of MZBs, defined as CD121^hi^CD23^lo^ B cells by flow cytometry, under various patho-physiological conditions, including normal BALB/c and B6 mice without or with LPS exposure (Fig. 1B, 7B), anti-DNA^Tg^ NZW mice (Fig. 6A), and autoimmune-prone NZB/NZW F1 and NOD mice that exhibit spontaneous MZB expansion (Fig. 6B,C, 7B). Similar observations were made using other markers for MZBs, including CD1d^hi^CD21^hi^ or CD1d^hi^CD9^+^ on gated CD19^+^ cells, in B6, NOD (Fig. 7B) and BALB/c mice (data not shown). Further evidence of reduction in MZBs *in vivo* was seen by immunohistochemistry showing reduction of B cells in marginal zones of spleen of BALB/c, B6 and NOD mice treated with αGalCer.

The MZ subset of B cells exhibits several unique features [34]. In the absence of B cell influx from bone marrow, FoBs in the spleen gradually decrease in numbers whereas MZBs are maintained at normal levels [35]. MZBs do not circulate via blood or lymph, and remain in spleen even after depletion of recirculating cells [36]. MZBs are more efficient than FoBs to generate plasma cells after polyclonal *in vitro* stimulation [37]. Findings in this paper provide evidence for a mechanism whereby such rapid and potent immune responses elicited by MZBs can be regulated by iNKT cells that can also respond and act rapidly.

The iNKT cells are a unique T cell subset that responds rapidly to lipid antigens [8], [9], [10]. iNKT cells efficiently promote B cell proliferation and Ig production [15], [16], [17]. Consistent with these observations, we found that iNKT cells enhance activation markers and costimulatory molecules on all B cells (Fig. 1). However, iNKT cells appear to regulate the two major subsets of splenic B cells differently. Whereas FoB cells expand and proliferate in presence of iNKT cells, iNKT cells selectively restrict the proliferation and numbers of MZBs.

We show evidence for at least two mechanisms, namely AICD and suppression of proliferation, whereby iNKT cells can control the homeostasis of MZBs. Cellular and molecular contexts in which these two cells interact are unclear. It has been proposed that MZBs that express high levels of CD1d will efficiently activate iNKT cells [38]. In fact, a recent study showed that MZBs activate iNKT cells *in vivo* and *in vitro* [39]. iNKT cells can in turn recognize CD1d on MZB cells and activate them. Activated iNKT cells also express enhanced levels of Fas-L [40], which could engage Fas on activated MZBs leading to their apoptosis. Such feedback inhibitory loop may ensure a homeostasis of these two immune cells after an incidence of rapid cross-activation. In support of this idea, we show that iNKT cells potently inhibit MZB cells if they express CD1d. CD1d expression on MZB or MZB-like cells has also been shown to promote iNKT cell-dependent tolerance [41] and protection from inflammation [42] by facilitating iNKT–B cell interactions. Thus, CD1d-restricted regulation of B cells may be an important mechanism whereby iNKT cells might discriminate between MZB versus other B cells leading to their selective regulation versus activation depending on the context.

iNKT cells can convert tolerogenic B cells into immunogenic APCs that can induce CD8^+^ Treg and cytotoxic T cells [43], [44], which can potentially regulate autoreactive B cells [45], [46]. However, our data show that iNKT cells can directly confer the regulatory effects on MZBs by themselves, without help from other immune cells. A previous study showed that F4/80^+^ APCs release a chemokine MIP-2 that recruits iNKT cells to the marginal zones of spleen where they can aggregate with MZBs and T cells for at least 7 days [44]. Such close interaction between iNKT cells and MZBs in splenic marginal zone may result in persistent activation of MZBs, ultimately leading to their AICD. Fas-mediated apoptosis is an important negative checkpoint during B cell development to eliminate autoreactive B cells and control B cell homeostasis [47]. We propose that Fas-mediated apoptosis induced by iNKT cells provide an important negative checkpoint at the level of mature MZBs. Since iNKT cells possess cytotoxic abilities [48], they may potentially reduce MZBs by killing them. However, we have so far found no evidence of iNKT cell-mediated direct killing of MZBs (our unpublished data). Although, iNKT cell-mediated inhibition of MZB cells was more potent when the two cells were in contact, some inhibition of MZBs occurred in a contact-independent manner. Cytokines that mediate this inhibition remain to be determined. A recent study has shown that upon stimulation with TLR ligands, peritoneal B1 B cells secrete high levels of IL-10 that then inhibits the proliferation of the same B1 B cells in an autocrine manner [49]. Interestingly, we have recently reported that iNKT cells reduce the numbers of IL-10–secreting B cells [28]. Thus, it would be interesting to investigate whether iNKT cells directly or indirectly inhibit IL-10–producing MZB cells [50], whereas IL-4 produced by iNKT cells might promote the activation and proliferation of FoBs [13]. Alternatively, iNKT cells might affect factors such as Notch2 or its ligands, BAFF or its receptors, chemotactic signals such as sphingosine 1 phosphate receptor (S1pR1), and integrins, which are known to promote the development, differentiation, migration and splenic retention of MZBs (reviewed in [51]).

Studies have linked MZB cell abnormalities to the development of autoimmune diseases [20], [21], [22], [24]. For example, MZB cells increase in lupus-prone [25], [26] and NOD mice prior to the onset of T1D [23], [24]. B cells with anti-self B cell receptors are enriched in the MZ [20], [27]. MZB is also the most potent B cell subset to activate naïve CD4 T cells [5]. MZB's ability to present self-antigens to autoreactive T cells, such as diabetogenic T cells [24], suggests a role of MZBs in the pathogenesis of autoimmune diseases. Interestingly, the MZB expansion correlates with the reduced iNKT cell numbers and functions in NOD mice [31] (Fig. 7). Impairment in iNKT cells prior to the onset of disease is a feature of many animal models of autoimmune diseases, including MRL-lpr and pristane-induced lupus [26], [52]. Reduction in NKT cells also associate with autoimmunity in the relatives of lupus patients [53]. Furthermore, germline deficiency of iNKT cells is associated with an expansion of MZB cells in aged mice [54], and CD1d-deficient BALB/c mice injected with a hydrocarbon oil that induces lupus-like disease have more MZB cells than CD1d^−/−^ BALB/c mice injected with PBS [26]. Taken together, it is reasonable to speculate that iNKT cell impairment might be related to MZB expansion in autoimmune conditions.

We provide a direct evidence for regulatory interactions between the two innate immune cells in autoimmunity that iNKT cell activation reduced MZBs in lupus- and autoimmune diabetes-prone mice (Fig. 6 and 7), and specifically inhibited ‘autoreactive’ (anti-DNA) MZB cells (Fig. 6A). In fact, iNKT cells can inhibit autoantibody production, while generally enhancing the normal antibody response in lupus-prone mice [28]. Similar observations have been reported in another model where injections of syngeneic apoptotic cells transiently trigger autoantibody production. In this model of induced autoantibody production, the absence or reduction of iNKT cells leads to increased autoreactive B cell activation without affecting the activation of B cells reactive to NP-OVA [55]. Importantly, αGalCer treatment prevents autoimmune disease in many experimental models including NOD mice [32], [33]. Such protective effect is believed to be mediated via promotion of Th2 responses [32], [33]. However, protection from diabetes in NOD mice is still maintained if iNKT cells are unable to produce or induce IL-4 and other regulatory cytokines (reviewed in [56]). Although, the role of MZBs in the pathogenesis of T1D is not proven yet, reduction of autoreactive MZBs in αGalCer-treated NOD mice should be investigated as an alternative mechanism whereby αGalCer affords protection in T1D.

In summary, iNKT cells regulate homeostasis of MZBs that rapidly respond to blood-borne pathogens and potently activate autoimmune T cells. We provide evidence for AICD and CD1d-restricted inhibition of proliferation as two mechanisms whereby iNKT cells regulate MZB homeostasis. Understanding these mechanisms will open avenues for manipulation of MZBs in human autoimmune diseases, since patients with T1D and SLE and their family members have reduced numbers and/or responses of iNKT cells [53], [56]. The iNKT cell-based manipulation of immune responses is particularly appealing, given the limited polymorphism in CD1 genes [8], obviating one of the major hurdles of therapies aimed at highly polymorphic MHC class I and II system. Enhancing this appeal is our finding that iNKT cells differentially regulate different B cell subsets, thus allowing a selective manipulation of certain B cell functions.
